# East‐West Convergence in Oncology: Treating Different Diseases With the Same Therapy

**DOI:** 10.1002/cai2.70051

**Published:** 2026-02-16

**Authors:** Fei Ma, Yuchen Jiao

**Affiliations:** ^1^ Department of Medical Oncology, National Cancer Center/National Clinical Research Center for Cancer/Cancer Hospital Chinese Academy of Medical Sciences and Peking Union Medical College Beijing China; ^2^ State Key Laboratory of Molecular Oncology, National Cancer Center/National Clinical Research Center for Cancer/Cancer Hospital Chinese Academy of Medical Sciences and Peking Union Medical College Beijing China

**Keywords:** Eastern wisdom, evidence‐based medicine, modern oncology, traditional Chinese medicine

**Abbreviations**
CAE: Chinese Academy of Engineering
HER2: human epidermal growth factor receptor 2
TCM: traditional Chinese medicine

In the course of millennia, traditional Chinese medicine (TCM) has crystallized eastern medical wisdom into a pragmatic, patient‐centered worldview during clinical practice. Over the past century, western medicine has increasingly drawn theory support from TCM's conceptual framework, helping to accelerate the shift from empirical medicine to precision medicine. The earliest extant TCM classic, the Huangdi Neijing (Yellow Emperor's Classic of Internal Medicine), compiled during the Spring and Autumn Period (770–476 bce) and the Warring States Period (475–221 bce), articulates a principle that still resonates today: governing the many through the One.

This convergence is also philosophical. Taoism traces all things to a common source—“the Tao gives birth to the One; the One generates the Two, the Two generate the Three, and from the Three arise all things.”—while Confucianism embraces “harmony without uniformity,” the search for unity within diversity. In oncology, that means looking past outward differences to the inner drivers of disease. In clinical practice, this becomes the “yi bing tong zhi” theory, that is, treating different diseases with the same therapy. While tumors arise in different organs and present different signs and symptoms in clinical practice, their underlying imbalances may be due to blood stasis or deficiency of vital qi. On that common foundation, practitioners apply shared therapeutic principles—regulating the spleen and stomach, clearing heat and resolving toxins—to restore systemic balance and halt disease progression. Academician of Chinese Academy of Engineering (CAE), Sun Yan, a leading authority of clinical oncology in China, has often recited the TCM tenet of “treating different diseases with the same therapy.” Clinical breakthroughs in modern oncology's precision targeted therapy echo this concept, bridging time and space to connect eastern and western medicine in a shared exploration of life and health.

With the progress of Human Genome Project and the rise of comprehensive molecular profiling, our insight into cancer has moved from the macro to the micro. Tumors historically defined by various organs can harbor the same driver alterations. HER2 (human epidermal growth factor receptor 2) overexpression is observed in both breast and gastric cancers; HER2 mutations appear in subsets of lung cancers; alterations in HER2 signaling are found in portions of colorectal and gynecologic malignancies. In essence, tumors from different systems may share a common disease etiology—the western analogue to TCM's notion of shared pathogenesis. This insight has reshaped modern therapeutic practice.

Trastuzumab is the first targeted drug, which transformed outcomes in HER2‐positive breast cancer and subsequently prolonged survival in HER2‐positive advanced gastric cancer. A new generation of HER2‐targeted agents—exemplified by trastuzumab deruxtecan—leverages the precision of an antibody‐drug conjugate and a distinctive bystander effect to deliver benefit across multiple HER2‐altered tumors, including breast, gastric, lung, and colorectal cancers. In practical terms, “one drug for many tumors” has moved from a conceptual framework to clinical reality. While western medicine anchors treatment on a shared molecular target, TCM centers its approach on a shared pathogenesis. The routes differ, but both aim at the common pathological essence—bringing the concept of treating different diseases with the same therapy from traditional experience onto the stage of modern evidence‐based medicine.

At the beginning of 2026, the editorial of Cancer Innovation is inviting leading oncologists across specialties to summarize global advances in diagnosis and treatment. The goal is to apply the principle of “treating different diseases with the same therapy” while balancing “seeking common ground and respecting differences.” This dialogue merges TCM's holistic view with western precision medicine, transforming an ancient concept into a modern, collaborative practice. This provides a deep insight for solving the complexity of tumor treatment.

On January 24, 2026, we mourned the passing away of Academician of CAE, Sun Yan at 96—a profound loss for oncology field. His eastern philosophical wisdom, scholarly integration of medical traditions, and visionary foresight provided essential frameworks for advancing cancer care. Looking forward, the integration of such wisdom, will ignite the light of hope “Treating different diseases with the same therapy” for tumor patients worldwide.

## Author Contributions

**Fei Ma:** project administration, supervision, writing – review and editing, conceptualization. **Yuchen Jiao:** writing – original draft, conceptualization.

## Funding

The authors received no specific funding for this work.

## Ethics Statement

The authors have nothing to report.

## Consent

The authors have nothing to report.

## Conflicts of Interest

Professor Fei Ma is a member of the *Cancer Innovation* Editorial Board. To minimize bias, he was excluded from all editorial decision‐making related to the acceptance of this article for publication. The remaining author declares no conflict of interest.

## Data Availability

The authors have nothing to report.

